# Oldest North American orthostern scorpion from the Carboniferous (Pennsylvanian: Bashkirian) Pottsville Formation of Alabama, USA

**DOI:** 10.1007/s00114-026-02180-6

**Published:** 2026-10-27

**Authors:** Gen A. Conway, Russell D. C. Bicknell, Bruno A. Buzatto, Adiël A. Klompmaker

**Affiliations:** 1 College of Science and Engineering, Flinders University, Adelaide, South Australia GPO 2100 Australia; 2 Division of Paleontology (Invertebrates), American Museum of Natural History, New York City, NY USA; 3 The University of Alabama Museums Research & Collections, The University of Alabama, Tuscaloosa, AL 35487 USA

**Keywords:** Scorpion, Palaeontology, Carboniferous, Arachnid, Orthosterni

## Abstract

Scorpions are an ancient chelicerate group with a fossil record extending back to the Silurian and a peak in reported fossil forms during the Carboniferous. Most Carboniferous scorpions are from Coal Measures sites, a small number of which exceptionally preserve evidence of respiratory structures. To expand the record of such material, we present a new fossil scorpion specimen from the Pennsylvanian (Bashkirian) Pottsville Formation from the Union Chapel Mine, Alabama, USA. We demonstrate that UV imagery is a useful tool for examining fossil scorpions, as it can clearly illustrate exoskeletal sections, and other important morphological structures. This specimen shows probable intrasternal spiracles, illustrating this is one of the oldest members of the derived scorpion infraorder Orthosterni, and potentially preserves evidence of book lungs. Due to the lack of taxonomically important features, such as coxosternal morphologies, we cannot identify the specimen beyond Orthosterni. We used Energy Dispersive X-Ray Spectroscopy (EDS) to explore fossil preservation, determining the specimen is preserved almost entirely as carbon, indicating it had undergone kerogenisation. This body fossil complements walking trace fossils from the same site attributed to scorpions, represents the first fossil scorpion from the southeastern USA, and provides more evidence for extensive diagenetic alteration of cuticle in fossil scorpions from Coal Measures.

## Introduction

Scorpions are a morphologically diverse (Dunlop and Harms 2026) arachnid order with chelate pedipalps, a venomous stinging telson, and pectine sensory organs (Howard et al. 2019). Today they are generally nocturnal and often fossorial (Polis 1990). There are currently 2918 extant (Prendini 2026) and ~ 170 extinct described species of scorpion (Dunlop and Garwood 2024; Prendini 2026). Scorpions are the oldest arachnids in the fossil record, with records extending to at least 435 Mya (mid-Silurian) (Laurie 1899; Wolfe et al. 2016; Howard et al. 2019). They potentially represent the first fossilised terrestrial animals (Callaghan and Boyce 2025), though whether the first scorpions were aquatic or terrestrial is still debated (Howard et al. 2019; Dunlop and Garwood 2024; Callaghan and Boyce 2025).

Palaeozoic scorpions were much more morphologically disparate than extant scorpions (Kjellesvig-Waering 1986), with particularly high morphological and taxonomic diversity during the Carboniferous (Dunlop and Garwood 2024). Molecular phylogenies frequently obtain a crown group divergence around the Carboniferous (Sharma et al. 2018; Howard et al. 2019, 2020; Lozano-Fernandez et al. 2020; Loria et al. 2022), reflecting the importance of this period for scorpion evolution. Indeed, the first unequivocal orthosterns—scorpions with the modern configuration of rectangular sternites with intrasternal, rather than marginal, spiracles—appeared by the Pennsylvanian (Vogel and Durden 1966; Jeram 1994a).

Whether the apparent Carboniferous scorpion diversity is genuine, or preservational bias from an exceptional fossil record afforded by the Coal Measures is an ongoing discussion (Legg et al. 2012; Dunlop and Garwood 2024). Further, while there is a definite signal of increased diversity from European and North American Coal Measures, historic excessive splitting has also contributed to particularly high species counts (Dunlop et al. 2016; Poschmann et al. 2016; Dunlop and Garwood 2024). Due to these issues, there are numerous monospecific genera and families (Dunlop and Garwood 2024), as well as many species known from a single specimen (Kjellesvig-Waering 1986). To expand these records, we report a new fossil scorpion from the Pennsylvanian (Bashkirian) of Alabama, USA, discuss the systematic position of the material, and the pathway to preservation.

## Geological context

**Fig. 1:**
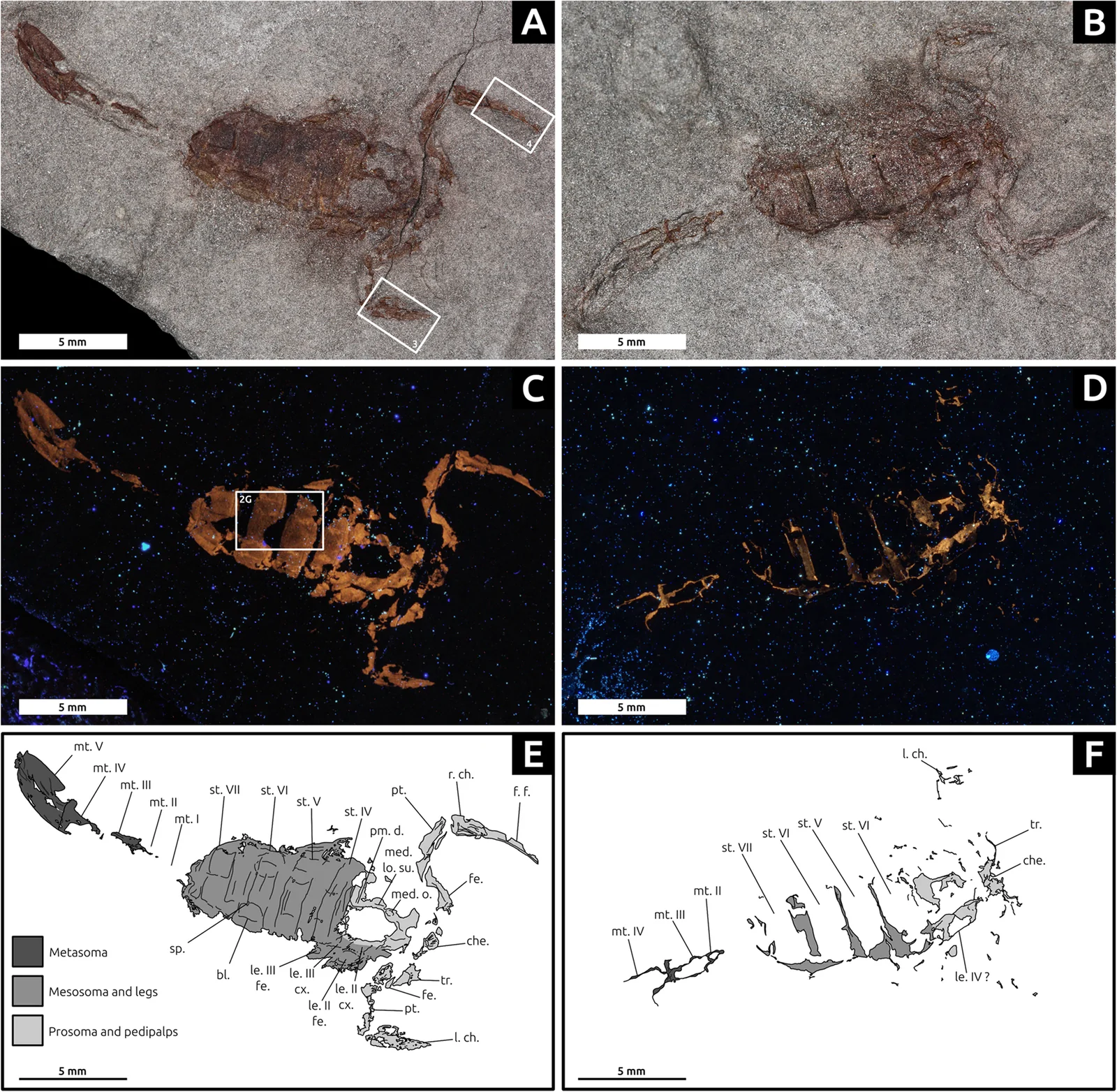
Part and counterpart of the fossil scorpion attributed to Orthosterni from the Pennsylvanian (Bashkirian) Pottsville Formation of the Union Chapel Mine, Alabama, USA. (**A**, **C**, **E**) UAMRC: Paleo:21967, part. (**A**) LED lighting. Boxes indicate areas examined with EDS. (**C**) UV lighting. (**E**) Interpretive drawing of part. (**B**, **D**, **F**) UAMRC: Paleo:21967, counterpart. (**B**) LED lighting. (**D**) UV lighting. (**F**) Interpretive drawing of counterpart. Only one set of book lungs and spiracles labelled. Abbreviations: le.: leg; mt: metasoma; st.: sternite, med. o.: median ocelli; pt.: patella; ch.: chela; f. f.: fixed finger; fe.: femur; che.: chelicerae; tr.: trochanter; cx.: coxa; bl.: book lungs; sp.: spiracles; pm. d.: posteromarginal depression; med. lo. su.: median longitudinal sulcus; l.: left in life; r.: right in life; I—VII: mesosomal tergite numbers

The part (Fig. 1B) and counterpart (Fig. 1A) of the specimen were collected loose on the surface on 30 April 2022 and 23 April 2022, respectively, at the Union Chapel Mine (also known as the Steven C. Minkin Paleozoic Footprint Site, UAMRC loc. 7), Walker County, Alabama. As almost all fossils from the site are found in mine spoil piles (Pashin 2005), a precise stratigraphic placement within the succession of the Pottsville Formation is impossible (Rindsberg et al. 2005; Beckemeyer and Engel 2011), but all specimens cited herein are from the upper part of the Pottsville Formation. Most fossils are from the dark grey shale layers, the so-called ‘*Cincosaurus* beds’ (Pashin 2005). The lower 4 m of the *Cincosaurus* beds contain pinstripe bedded mudstone and fossil tetrapod trackways, above which is 1 m of ripple-bedded sandstone and shale, and then 1 m of sandy underclay and lycopod root traces (Pashin 2005). The *Cincosaurus* beds sit between the overlying New Castle coal bed and underlying Mary Lee coal bed (Pashin 2005). The Union Chapel Mine site also exposes the New Castle roof shale—a 2.7 m thick grey silty shale overlying the New Castle coal bed that contains abundant plant fossils (Pashin 2005).

During the Pennsylvanian (Bashkirian), the Union Chapel Mine site was located in a humid tropical region near the palaeoequator, ~10° S (Pashin 2005). The *Cincosaurus* beds are interpreted as a tidally impacted estuarine mudflat, which animals ventured onto to forage (Pashin 2005). Abundant, well preserved, and articulated allochthonous plant fossils and vertebrate trackways indicate that the beds preserve both evidence of animals that lived at the depositional location and evidence from organisms in the surrounding area. The abundant plant fossils preserved in standing life position in the New Castle roof shale suggests this layer was deposited in a terrestrial swamp with occasional flooding (Pashin 2005).

The Union Chapel fossil site preserves an array of body, trace, and plant fossils. There are abundant tetrapod (Haubold et al. 2005; Hunt et al. 2005), xiphosuran (Buta et al. 2005; King et al. 2019), and myriapod trackways (Buta et al. 2005; Lucas and Lerner 2005), fish trace fossils (Hunt et al. 2005), and insect burrows known from the site (Buta et al. 2005; Rindsberg and Kopaska-Merkel 2005). Plant fossils include Lepidodendrales, Equisetales, Pteridosperms, and Cordaitales (Dilcher et al. 2005). While arthropod body fossils are rare from Union Chapel Mine (Atkinson 2005), wings of Palaeodictyoptera, Ephemeropterida, and Odonatoptera (Beckemeyer and Engel 2011, 2018) have been reported. These wing impressions potentially preserve original or altered cuticle (Beckemeyer and Engel 2011). Abundant plants are preserved within the shale layers (Dilcher et al. 2005). While rare, a nodular limestone representing a transgression has resulted in the preservation of marine fossils such as brachiopods, bivalves, and shark teeth (Pashin 2005; Egli in press).

Plant fossil biostratigraphy has dated the Pottsville Formation to the early Pennsylvanian (Butts and Adams 1926). Detailed pollen spores have further refined this to the Westphalian A local substage (318.96–316.28 Ma) (Eble and Gillespie 1989; Eble et al. 1991; Laurin et al. 2026), which is within the Bashkirian global stage (323.4–315.2 Ma) (Cohen et al. 2025). Argon dating has placed the Pottsville Formation as early Pennsylvanian, though there are some layers dated as young as 310 Ma (Uddin et al. 2016).

## Methods and materials

###  Imaging

The specimen is housed within the palaeontology collection of The University of Alabama Museums Research & Collections (UAMRC), Alabama, USA, under the specimen number UAMRC:Paleo:21967 and is a part (Fig. 1B) and counterpart (Fig. 1A). The specimen was imaged at the American Museum of Natural History using an array of imaging techniques—plain and flash photography using a Canon EOS 60D camera body and an EF 100 mm macro lens; a Nikon Z 6II camera body with a Nikkor Z MC 105 mm macro lens; and then under UVA lighting with a Sony Micro Nikkor 55 mm camera and a LP415-52 UV block lens over the lens. Trackways possibly produced by a scorpion (UAMRC:Paleo:13020 and UAMRC:Paleo:13053) were imaged using a Google Pixel 3a under low angle sunlight at The University of Alabama.

###  Scanning electron microscopy

To examine the elemental composition of the specimen, scanning electron microscopy (SEM) coupled with energy-dispersive X-ray spectroscopy (EDS) was used for the part of UAMRC:Paleo:21967. The part was analysed uncoated under low-vacuum conditions at an accelerating voltage of 10 kV, a working distance of 12 mm, and a beam current of 13.5 nA, using a Hitachi S-4700 SEM at the AMNH to collect back-scattered electron (BSE) images. Elemental maps were acquired using a Bruker AXS Quantax 4010 detector at a resolution of 2560 × 1920 pixels.

### Systematic palaeontology

General terminology follows Hjelle (1990) and terminology for carinae follows Prendini (2026) and Soleglad and Fet (2015). Selected measurements follow Sissom et al. (1990), and were taken in ImageJ (Schneider et al. 2012).

## Results

### Systematic palaeontology

#### Order Scorpiones

C.L. Koch 1850

#### Suborder Neoscorpionina

Thorell and Lindström 1885

#### Infraorder Orthosterni

Pocock 1911

**Orthosterni** ***incertae sedis***

#### Examined material

UAMRC:Paleo:21967, part and counterpart

#### Preservation

Specimen preserved dorsal down as a compression fossil in light gray shale, lacking topographic relief. In particular, the prosoma is highly taphonomically distorted and clearly asymmetrical (Fig. 1A). The specimen is probably a moult due to extended chelae and somewhat curved body line (McCoy and Brandt 2009). The specimen was preserved on a bedding plane with invertebrate burrows, and no footprints were associated with the body fossil.

#### Description

For all the following see Table 1 for measurements. All orientations are reported as the specimen would be in life.

**Table 1:** Measurements of specimen.— indicates the measurement could not be derived

Section	Length (mm)	Width (mm)
*Prosoma*		
Carapace anterior	—	2.4
Carapace posterior	—	3.5
Carapace	2.6	—
Anterior margin to median eyes	0.5	—
Median eyes to posterior margin	1.5	—
Ocular diad	—	0.6
*Pedipalps*		
Pedipalps total	8.5	—
Femur length	1.9	0.9
Patella	2.1	0.7
Chela	4.5	0.8
Fixed finger (width measured at base)	2.9	0.6
Chela palm	1.7	—
*Walking Legs*		
Femur, appendage IV	2.3	0.8
*Mesosoma*		
Tergite I	0.5	3.5
Tergite II	0.6	3.8
Tergite III	0.8	4.3
Tergite IV	1.0	4.2
Tergite V	1.5	4.0
Tergite VI	1.2	4.2
Tergite VII (width anterior margin)	1.4	3.4
Sternite IV	1.3	3.0
Sternite V	1.6	3.6
Sternite VI	1.6	3.9
Sternite VII (width anterior margin)	1.7	3.2
*Metasoma*		
Metasoma I	1.5	1.5
Metasoma II	1.5	1.5
Metasoma III	1.5	1.6
Metasoma IV	1.9	1.6
Metasoma V	3.4	1.7

####  Dorsal prosoma

Carapace poorly preserved (Fig. 1A), with limited cuticle around margin and midline. Median longitudinal sulcus and posteromarginal depression preserved (See annotations Fig. 1E). Carapace subquadrate shape with curved lateral margins. Anterior margin has wide anterior notch. Frontal lobes have rounded anterior margins. Posteromarginal depression is triangular and extends towards prosoma-metasoma articulation. Posterior margin slightly convex. Cuticle along centre of prosoma with two circular areas not fluorescent under UV (Fig. 1C) considered median eye tubercle (See annotations Fig. 1E), median ocelli that are moderately sized, and located ~ 1/3 along prosoma. Lateral eyes are not preserved. However, prosomal shape is highly taphonomically distorted and clearly asymmetrical (Fig. 1A). These may not accurately represent morphology in life.

####  Ventral prosoma

**Fig. 2:**
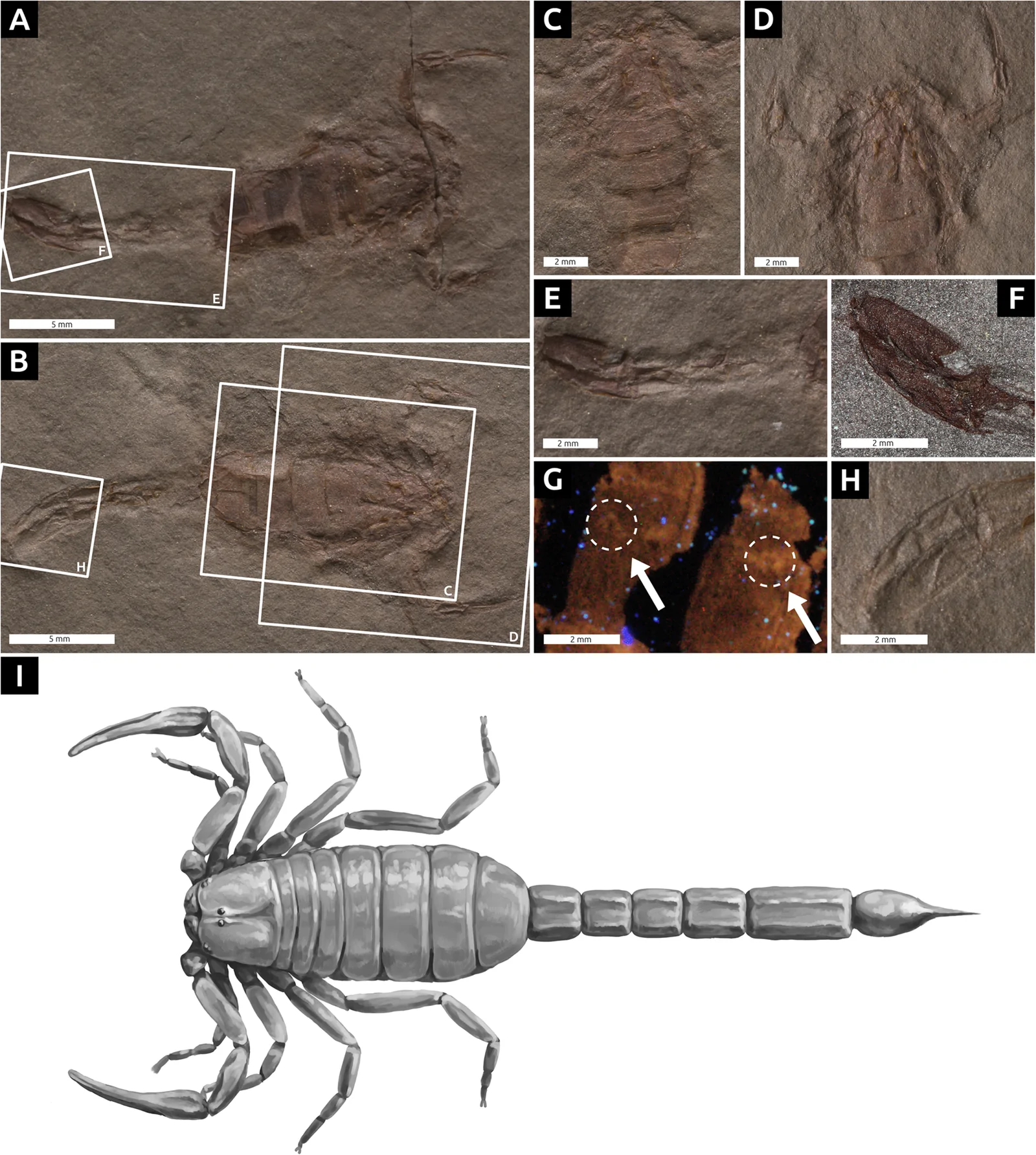
Closeups of specimen showing different directional lighting. (**A**) Part with low angled lighting showing inverse reliefs of prosoma + mesosoma and metasoma. (**B**) Counterpart with low angle lighting. (**C**) Closeup of mesosoma on counterpart showing impressions of boundaries between segments. (**D**) Closeup of mesosoma on counterpart showing femoral impressions. (**E**) Closeup of metasoma on part. (**F**) Closeup of metasoma V cuticle showing continuous cuticle without defined articulation. (**G**) Closeup of part under UV showing areas interpreted as spiracles (circles and arrows). (**H**) Impression of metasoma V on counterpart, showing potential carinae. (**I**) Reconstruction of scorpion; legs, telson and lateral eyes are speculative and carinae may be ventral

Ventral region very poorly preserved. Cuticle mostly absent (Fig. 1A) and any present in region that could potentially represent ventral prosomal features considered record of carapace. Linear structures under UV for counterpart (Fig. 1D) may represent coxapophyses or displaced fragments or carapace. Deep, diagonal impression in this region (Fig. 2D) indicative of sulcus along carapace. No evidence of pectines.

#### Chelicerae

Cuticle partially preserved in extended position. Chelae indistinct due to preservation.

#### Pedipalps

Both pedipalps partially preserved. Left pedipalp shows patchy regions of cuticle and proximal region of fixed finger. Right pedipalp better preserved, lacking complete cuticle preservation, only fixed finger present (Fig. 1A). Ridges along pedipalps may be carinae or taphonomic distortion. Ridges along pedipalp femur, patella considered taphonomic. Depression between manus and finger of right chelae interpreted as movable finger articulation. Prominent ridges and depressions visible on left chelae (Fig. 1A, C) interpreted as ventroexternal and ventromedian carinae. Ridge on fixed finger inner margin is prominent carina. Dentition not observed. Chelae gracile with narrow elongated fingers.

####  Walking legs

Right walking leg IV on counterpart fossil (see Fig. 2C, D). Potentially disarticulated leg segment anterior to main fossil. However, may also be metasomal cuticle, or tergal fragment.

####  Mesosoma

Mesosoma well preserved, with clear, overlapping sternite and tergite margins. Distinguishing between boundaries in part is complicated (Fig. 1A). As such, counterpart used (Fig. 2C). Anterior tergites shorter and narrower (Table 1) than posterior tergites that become longer posteriorly. Sternites preserved in darker material (Fig. 2A, B) that fluoresces under UV (Fig. 1C). Sternites IV–VII mostly intact, and right lateral portion of sternite III may be preserved (Fig. 1C).

Sternites IV–VI show two pairs of brightly fluorescent lines parallel to body (Figs. 1C and 2G), with medial lines curving laterally. Structures not present on sternite VII, indicating presence of book lungs. Same structures have darker spots ringed by brighter fluorescent cuticle (arrows in Fig. 2G) considered round, intramarginal respiratory spiracles. Prominent ridge on anterior margin of sternites IV–VI present. Posterior margin straight with no lobation. Sternite VII comparable length to preceding sternites (Table 1) with two darker lines visible (Fig. 1C) considered ventrosubmedian or ventrolateral carinae. Sternites do not overlap.

####  Metasoma 

The metasoma is moderately robust. Anterior metasoma preserved as impressions, while posterior region preserved as cuticle (Figs. 1A and 2E). Metasoma almost entirely preserved as impression on counterpart showing either articulations or carinae. Metasomal segments I–IV similarly sized and approximately same length as width. Precise margins uncertain for metasoma I–III (Table 1). Prominent, likely ventrolateral and paired ventromedian carinae visible on metasoma IV. Same structures less clear as impressions on metasoma I–III (Fig. 2A, B, E).

Metasoma V elongated compared to other metasomal segments and potentially flattened or rotated. Metasoma V on counterpart shows carinae with defined granulation (Fig. 2H), potentially ventral lateral carinae. Telson likely absent but potentially preserved folded over metasoma V.

###  Remarks

The classification often used for fossil scorpions primarily follows Kjellesvig-Waering (1986), Dunlop and Garwood (2024), and Prendini (2026). Within this framework, scorpions are separated into infraorders by six different abdominal plate shapes, considered the most important character associated with aquatic or terrestrial life modes. Families are differentiated by coxosternal and eye morphology (Kjellesvig-Waering 1986). These are mostly ventral features, limiting the application to specimens lacking this data. Further, Kjellesvig-Waering’s framework has been implicated in questionable interpretations of material (Jeram 1994b; Shear and Selden 1995; Dunlop et al. 2008; Kühl et al. 2012; Dunlop and Garwood 2024) that may have been impacted by taphonomic or ontogenetic variability (Jeram 1994b; Farley 2005; Dunlop et al. 2016), misalignment with extant scorpion taxonomy (Stockwell 1989), and excessive splitting (Fet et al. 2000; Dunlop et al. 2008; Legg et al. 2012; Dunlop and Garwood 2024). Synonymising currently differentiated fossil forms that likely represent the same species has since been highlighted as important (Dunlop and Garwood 2023). However, there are no recent comprehensive revisions of fossil scorpion systematics, and Kjellesvig-Waering (1986) is still used due to lack of alternatives. Combined with the incomplete preservation of the fossil, we opted to limit taxonomic assignment to a high rank.

Neoscorpionina is a group that contains morphologically modern scorpions and includes all forms with respiratory spiracles (Kjellesvig-Waering 1986), the posterior position of median eyes compared to the anterior margin position in more basal forms (Stockwell 1989), and elongated fifth metasomal segments (Jeram 1994a). The presence of respiratory spiracles and elongated fifth metasomal segment confidently place the Union Chapel Mine material within Neoscorpionina.

Neoscorpionina has since been split into two infraorders—Palaeosterni and Orthosterni—based on lateral eye, spiracle, median eye, and coxae morphologies (Stockwell 1989). Palaeosterni, erected by Stockwell (1989), is differentiated from Orthosterni by the presence of spiracles on the posterior margins of sternites, as opposed to centrolaterally, as observed in most fossil orthostern and all extant scorpions, as well as lateral eyes of eight or zero subunits. However, the presence of marginal spiracles in some Palaeosterni has been questioned (Jeram 1994a). Jeram (1994a) resolved more autapomorphies of Orthosterni—chelae dentition, pedipalp trichobothria and cuticle ornament—to the exclusion of Palaeosterni. However, these orthostern features are not preserved in this fossil. The markings on sternites that are visible under UV likely represent intrasternite spiracles (Fig. 2G), and as such, we place our material within Orthosterni.

Most characters defining fossil scorpion families and genera include lateral eyes, coxosternal morphology and carapace shape. Lateral eyes and coxosternal morphology are not preserved here. Further, while the carapace appears short and semicircular, this structure is distorted and only partially preserved, limiting the utility here (Legg et al. 2012). Consequently, we cannot confidently identify this specimen to a lower taxonomic group than Orthosterni or demonstrate that it belongs within a known species. However, we do note that our material is distinct from the other scorpion known from the Pottsville Formation, *Opsieobuthus pottsvillensis* Moore 1923. This species has distinctly lobed posterior margins of the sternites (Moore 1923; Kjellesvig-Waering 1986; Stockwell 1989), while our new specimen has rectangular sternites.

Fossil scorpions are very rare and usually poorly preserved (Jeram 1994b), with many species known from very few specimens (Table 2 for Carboniferous records from North America) and described with limited morphological interpretation (Jeram 1998). Consequently, many Palaeozoic scorpions were described from very incomplete material, with many Palaeozoic species based on isolated pedipalp claws or fingers (Kjellesvig-Waering 1954, 1972, 1986; Dunlop and Garwood 2023, 2024; Howard et al. 2026). With multiple species described from extremely incomplete material, finding grounds for either synonymy or demonstrating taxonomic uniqueness is difficult (Jeram 1994b). Further, most scorpion species are only known from either dorsal or ventral features (Jeram 1998; Dunlop et al. 2016). This is problematic for placing species into the current framework that uses almost exclusively ventral features (Kjellesvig-Waering 1986; Dunlop and Garwood 2024).

**Table 2:** Records of Carboniferous scorpions of North America showing presentation of major exoskeletal sections

Species	Author	‍Suborder (Infraorder)	Holotype	Prosoma	Coxosternal region	Pedipalps	Mesosoma dorsal	Mesosoma ventral	Metasoma dorsal	Metasoma ventral	Legs	Other material	Additional‍ key references	Locality	Geological unit	Age (Ma)	Global stage
*Eoscorpius casei*	Kjellesvig-Waering, 1986	Mesoscorpionina	PU 87266	Absent	Well preserved	Absent	Absent	Poorly preserved	Absent	Absent	Poorly preserved	—	Stockwell 1989	West Bay, Nova Scotia, Canada	Parrsboro Formation	318.7–316.9	Bashkirian
*Labriscorpio alliedensis*	Leary, 1980	‍‍*Incertae sedis*	ISM 416899	Absent	Well preserved	Well preserved	Well preserved	Absent	Absent	Absent	Poorly preserved	—	‍Kjellesvig-Waering,^1986^, Stockwell 1989; Jeram 1998	Allied Stone Company quarry, Illinois, USA	No formation determined, Allied Stone Company quarry	323.4–315.2	Bashkirian
Orthosterni *incertae sedis* (this specimen)		Neoscorpionina (Orthosterni)‍	UAMRC:Paleo:21967	Poorly preserved	Absent	Well preserved	Poorly preserved	Well preserved	Poorly preserved, ambiguous whether dorsal or ventral	Poorly preserved, ambiguous whether dorsal or ventral	Poorly preserved	—	—	Union Chapel Mine, Alabama, USA	upper Pottsville Formation	319.0–316.3	Bashkirian
*Opsieobuthus pottsvillensis*	Moore, 1923	Mesoscorpionina	FMNH (UC) 37984	Absent	Well preserved	Poorly preserved	Absent	Well preserved	Absent	Poorly preserved	Poorly preserved	—	Kjellesvig-Waering 1986, ‍Stockwell 1989; Jeram 1994b	Clay City, Indiana, USA	Pottsville Formation	318.7–314.6	Bashkirian– Moscovian
*Boreoscorpio copelandi*	Kjellesvig-Waering, 1986	‍*Incertae sedis*	GSC 12778	Poorly preserved	Poorly preserved	Poorly preserved	Poorly preserved	Poorly preserved	Well preserved	Well preserved	Poorly preserved	—	‍Stockwell 1989	Stellarton Coalfield, Nova Scotia, Canada	Pictou Group	314.6–307.4	Moscovian
*Buthiscorpius lemayi*	Kjellesvig-Waering, 1986	Neoscorpionina (Palaeosterni)‍	FMNH (PE) 21600	Well preserved	Well preserved	Poorly preserved	Well preserved	Well preserved	Poorly preserved	Poorly preserved	Poorly preserved	—	‍Stockwell 1989	Mazon Creek, Illinois, USA	Carbondale Formation	309.8–307.4	Moscovian
*Eoscorpius carbonarius*	Meek and Worthen, 1868	‍Mesoscorpionina	FMNH 8949	Well preserved	Absent	Poorly preserved	Well preserved	Absent	Poorly preserved	Absent	Poorly preserved	YPM IP 128, USNM 37977, USNM 37986, 4 additional specimens	Kjellesvig-Waering 1986, ‍Stockwell 1989	Mazon Creek, Illinois, USA	Carbondale Formation	309.8–307.4	Moscovian
*Eoctonus miniatus*	Petrunkevitch, 1913	Neoscorpionina (Palaeosterni)‍	YPM 131	Poorly preserved	Absent	Poorly preserved	Well preserved	Poorly preserved	Poorly preserved	Poorly preserved	Poorly preserved	FMNH 16498	‍Kjellesvig-Waering 1986; Stockwell 1989	Mazon Creek, Illinois, USA	Carbondale Formation	309.8–307.4	Moscovian
*Kronoscorpio danielsi*	Petrunkevitch, 1913	‍Mesoscorpionina	UMMP 7216	Well preserved	Well preserved	Poorly preserved	Well preserved	Poorly preserved	Absent	Absent	Poorly preserved	FMNH (PE) 32085	‍Stockwell 1989 Jeram 1994b; Miether and Dunlop 2016	Mazon Creek, Illinois, USA	Carbondale Formation	309.8–307.4	Moscovian
*Mazonia woodiana*	Meek and Worthen, 1868	‍Mesoscorpionina	FMNH (PE) 39253	Well preserved	Poorly preserved	Poorly preserved	Well preserved	Well preserved	Poorly preserved	Poorly preserved	Poorly preserved	One additional specimen	Kjellesvig-Waering 1986, ‍Stockwell 1989	Mazon Creek, Illinois, USA	Carbondale Formation	309.8–307.4	Moscovian
*Palaeobuthus distinctus*	Petrunkevitch, 1913	‍*Incertae sedis*	YPM IP 113	Poorly preserved	Poorly preserved	Absent	Poorly preserved	Poorly preserved	Poorly preserved	Poorly preserved	Poorly preserved	YPM IP 127, YPM IP 130, BU 721 A, B,C	‍Kjellesvig-Waering 1986; Stockwell 1989	Mazon Creek, Illinois, USA	Carbondale Formation	309.8–307.4	Moscovian
*Palaeopisthacanthus schucherti*	Petrunkevitch, 1913	‍Neoscorpionina (Orthosterni)‍	YPM 140	Poorly preserved	Well preserved	Well preserved	Well preserved	Well preserved	Absent	Absent	Poorly preserved	One additional specimen	‍Kjellesvig-Waering 1986, ‍Stockwell 1989; Jeram 1994a; Solegad & Fet 2003	Mazon Creek Illinois, USA	Carbondale Formation	309.8–307.4	Moscovian
*Paraisobuthus virginiae*	Kjellesvig-Waering, 1986	‍Mesoscorpionina	YPM 132	Well preserved	Well preserved	Poorly preserved	Well preserved	Poorly preserved	Poorly preserved	Well preserved	Absent	—	‍‍Stockwell 1989	Mazon Creek, Illinois, USA	Carbondale Formation	309.8–307.4	Moscovian
*Titanoscorpio douglassi*	Kjellesvig-Waering, 1986	‍*Incertae sedis*	Private collection	Absent	Absent	Absent	Absent	Absent	Absent	Absent	Poorly preserved	Two additional specimens, in private collection	‍‍Stockwell 1989	Mazon Creek, Illinois, USA	Carbondale Formation	309.8–307.4	Moscovian
*Telmatoscorpio brevipectus*	Kjellesvig-Waering, 1986	‍Mesoscorpionina	YPM 129	Well preserved	Well preserved	Poorly preserved	Well preserved	Poorly preserved	Poorly preserved	Absent	Absent	—	‍Stockwell 1989	Mazon Creek, Illinois, USA	Carbondale Formation	309.8–307.4	Moscovian
*Corniops mapesii*	Jeram, 1994a	‍Neoscorpionina (Orthosterni)‍	BELUM 14082	Absent	Absent	Poorly preserved	Absent	Absent	Absent	Absent	Absent	—		Lone Star Lake, Kansas, USA	Lawrence Shale Formation	307.0–303.7	Kasimovian
*Cryptoscorpius americanus*	Jeram, 1994a	Neoscorpionina (Orthosterni)‍	BELUM 14083a	Absent	Absent	Poorly preserved	Absent	Absent	Absent	Absent	Absent	BELUM 14083b, BELUM 14079, BELUM 14080, BELUM 14081		Lone Star Lake, Kansas, USA	Lawrence Shale Formation	307.0–303.7	Kasimovian
*Garnettius hungerfordi*	Elias, 1936	‍*Incertae sedis*	USNM 12543	Well preserved	Absent	Well preserved	Well preserved	Poorly preserved	Poorly preserved	Poorly preserved	Well preserved	—	Kjellesvig-Waering 1986, ‍Stockwell 1989	Garnett, Kansas, USA	Stanton Formation	307.0–303.7	Kasimovian
*Palaeopisthacanthus vogelandurdeni*	Jeram, 1994a	‍Neoscorpionina (Orthosterni)‍	BELUM 14086	Absent	Absent	Poorly preserved	Absent	Absent	Absent	Absent	Absent	—	‍—	Lone Star Lake, Kansas, USA	Lawrence Shale Formation	307.0–303.7	Kasimovian

Ordered by stage and then alphabetical by genus. Infraorders follow Stockwell/Jeram classification. Institutional abbreviations: BELUM: Ulster Museum, Belfast, Northern Ireland, UK; BU: Birmingham University, Birmingham, West Midlands, UK; FMNH: Field Museum of Natural History, Chicago, Illinois, USA; GSC: Geological Survey of Canada, Ottawa, Ontario, Canada; ISM: Illinois State Museum, Springfield, Illinois, USA; PU: Princeton University, Princeton, New Jersey, USA; UMMP: University of Michigan Museum of Palaeontology, Ann Arbor, Michigan, USA; USNM: Smithsonian National Museum of Natural History, Washington, D.C, USA; YPM IP: Yale Peabody Museum, New Haven, Connecticut, USA. Formation data and records of additional material from the Palaeobiology Database, 2026

Carboniferous scorpions have commonly been described as a new species using singular specimens (Poschmann et al. 2016). Fortunately, revisions Coal Measures sites over recent decades have substantially reduced the number of species by recognising synonyms (Legg et al. 2012; Dunlop et al. 2016; Dunlop and Garwood 2024); for example, *Compsoscorpius buthiformis* (Pocock 1911) has nine junior synonyms (Legg et al. 2012). Many scorpions described as different species likely represent an ontogenetic grade (Dunlop et al. 2008)—an important direction for accurately reconstructing the fossil record and evolution of the group.

## EDS Results

**Fig. 3:**
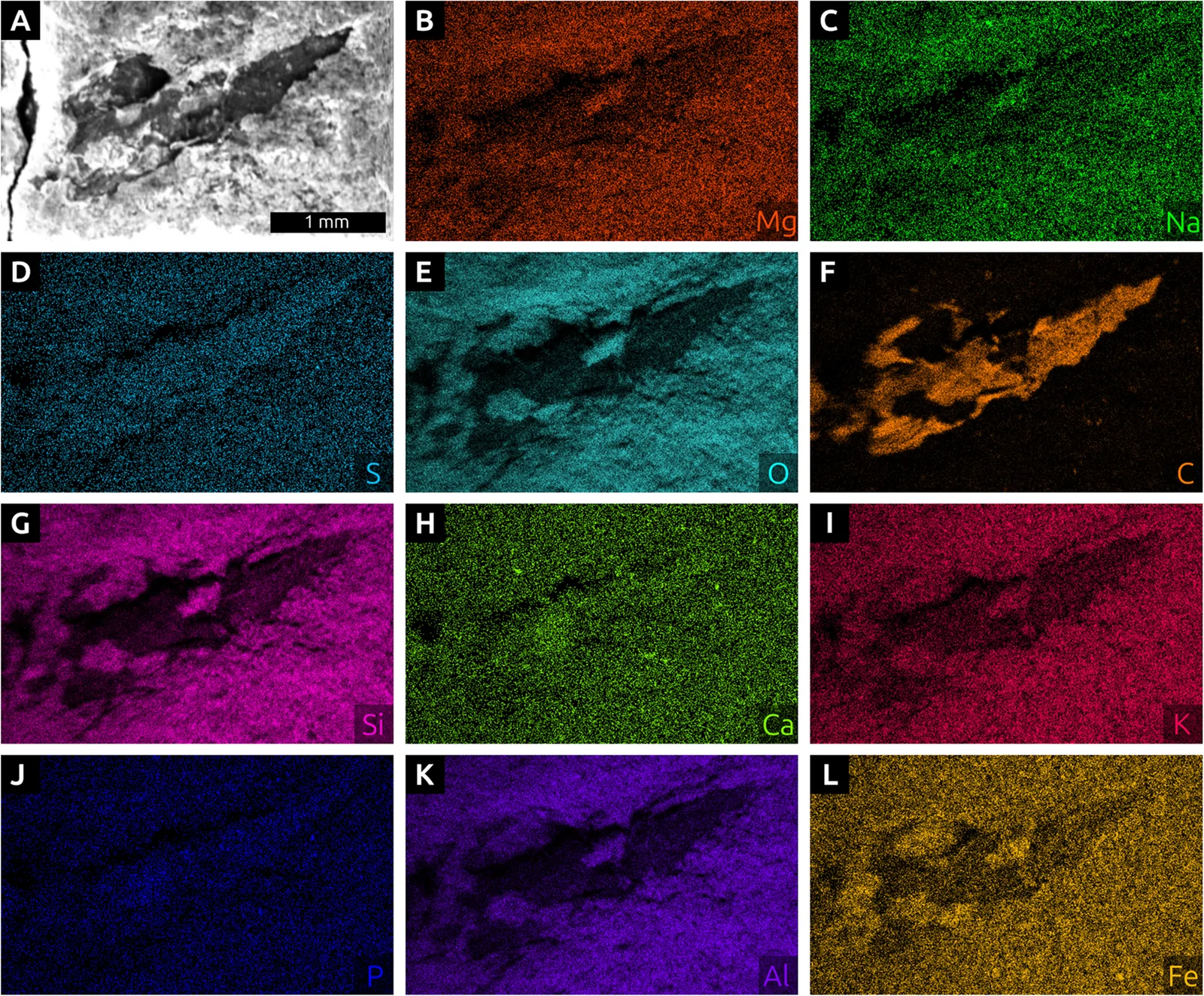
SEM backscatter image and EDS elemental maps of left chela, area indicated in Fig. 1. (**A**) Backscatter image of chela. (**B**–**L**) Elemental maps. (**B**) Magnesium. (**C**) Sodium. (**D**) Sulphur. (**E**) Oxygen. (**F**) Carbon. (**G**) Silicon. (**H**) Calcium. (**I**) Potassium. (**J**) Phosphorus. (**K**) Aluminium. (**L**) Iron. Scale bar in (**A**) applicable to all other images

**Fig. 4:**
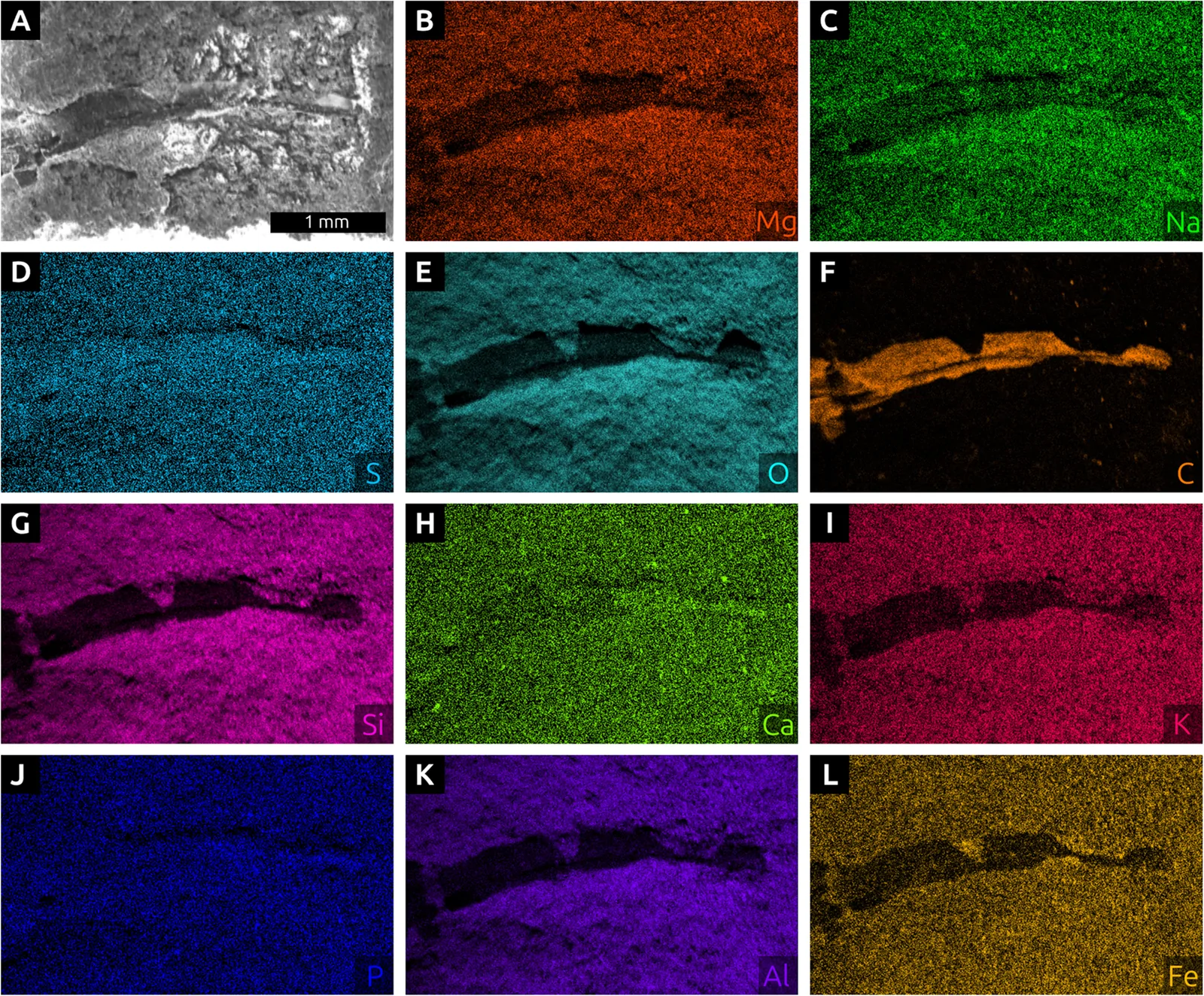
SEM backscatter image and EDS elemental maps of right chela, area indicated in Fig. 1. (**A**) Backscatter image of chela. (**B**–**L**) Elemental maps. (**B**) Magnesium. (**C**) Sodium. (**D**) Sulphur. (**E**) Oxygen. (**F**) Carbon. (**G**) Silicon. (**H**) Calcium. (**I**) Potassium. (**J**) Phosphorus. (**K**) Aluminium. (**L**) Iron. Scale bar in (**A**) applicable to all other images

Elemental dispersive spectroscopy of the left chela (Fig. 3) and the distal portion of the right chela (Fig. 4) evidence enrichment and depletion of different elements in the fossil compared to the matrix. The fossil shows considerable enrichment in carbon, whereas the matrix shows an absence of this element (Figs. 3F and 4F). Magnesium, sodium, oxygen, silicon, potassium, aluminium and iron are depleted in the fossil (Figs. 3B, C, E, G, I, K and L and 4B, C, E, G, I, K and L). Sulphur, calcium and phosphorus are present in similar concentrations in the fossil and the matrix (Figs. 3D, H and J and 4D, H and J). There is substantial enrichment in oxygen, aluminium and silicon and moderate enrichment of potassium and iron in the matrix surrounding the fossil.

## Discussion

The Union Chapel Mine scorpion represents the first record of Orthosterni within the early Pennsylvanian (Bashkirian) of North America (Table 2), the earliest known North America orthostern scorpion, and the first fossil scorpion from the southeastern USA. However, early orthostern records are known from Europe. The only other equivalently early complete orthostern scorpion is *Compsoscorpius buthiformis* (Pocock, 1911) also from Bashkirian (Westphalian A–B) sites in the British Midlands in the United Kingdom (Legg et al. 2012). Orthostern-like cuticle fragments are also present from the Mississippian (Visean) of East Kirkton Quarry, West Lothian, Scotland (Jeram 1994b). This record herein is therefore useful for constraining the date of morphologically modern scorpions for phylogenies.

**Fig. 5:**
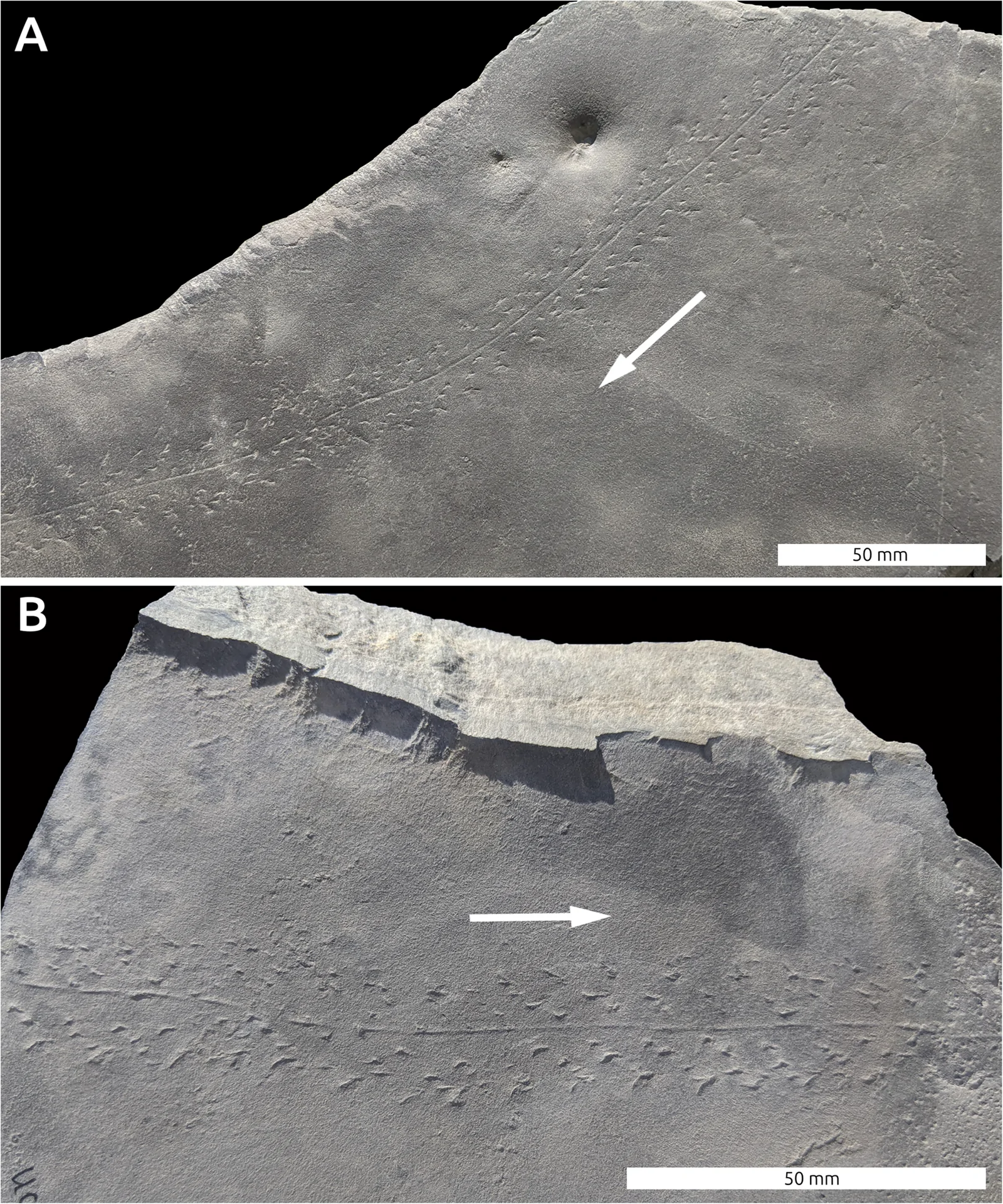
Trackways from the Union Chapel Mine attributed to a possible scorpion tracemaker (Buta and Kopaska-Merkel 2016). (A) UAMRC:Paleo:13053. (B) UAMRC: Paleo:13020. Arrows indicate likely direction of movement based on Sadler (1993, Fig. 5.2).

 The body fossil supports the interpretation of traces from the same site previously attributed to scorpions (Fig. 5; UAMRC:Paleo:13020, 13053, 13076, 13084(=13085), 24346–24353). Buta & Kopaska-Merkel (2016, p. 231–232, Fig. 16.13) argued that multiple trackway specimens resembling *Octopodichnus* Gilmore, 1927 and *Paleohelcura* Gilmore, 1926 might be produced by a scorpion tracemaker (see Sadler 1993; Seilacher 2007; Davis et al. 2007; Clendenon and Brand 2023), though extant scorpions usually do not leave tail drag marks (Sadler 1993; Davis et al. 2007). Additionally, trackways classified of *Stiaria* Smith, 1909 are also known from the site (Buta & Kopaska-Merkel 2016, Figs. 9.4, 16.1, 16.3) and have been linked to scorpions (Lucas et al. 2013). The examined specimen measures ~ 21.9 mm in length and 4 mm in width, whereas examined trackways are around ~15–22 mm wide (Fig. 5). Given lack of data on leg splay, we cannot confidently conclude if an individual of comparable size to our specimen produced these traces. Further, given the range of trace widths it seems reasonable that these traces reflect different size classes of co-occurring scorpions, if these are scorpion traces.

###  Exceptionally preserved morphology

Lung spiracles, or stigmata, are external openings of book lungs allowing intake of air and are observed on modern scorpions and select Carboniferous orthosterns (Jeram 1994a). The presence of these structures within a Devonian scorpion has been used to suggest the deep origin of terrestrial respiratory organs (Kühl et al. 2012). The Union Chapel Mine fossil also shows evidence of these structures (Fig. 2G). *Palaeopisthacanthus schucherti* Petrunkevitch, 1913 from the Mazon Creek Lagerstätte (Pennsylvanian [Moscovian], Illinois, USA) (Vogel and Durden 1966) and *Compsoscorpius buthiformis* (Pocock, 1911) (Pennsylvanian [Bashkirian-Moscovian] Coseley Lagerstätte, West Midlands, England) (Legg et al. 2012) both preserve circular spiracles in comparable locations to our material. Similar to book lungs, spiracles would be expected in many fossil scorpions but are rarely described, and their anatomy may be more variable in fossil species (e.g. potential marginal spiracles).

Curved, paired lateral lines on all but the posteriormost sternite are suggestive of book lungs (Scholtz and Kamenz 2006; Haug et al. 2019) (Fig. 1C). Despite the moulted condition, book lungs remain after the exoskeleton is shed (Gaban and Farley 2002). The fossil record of Paleozoic book lungs is limited, with unequivocal evidence in *Pulmonoscorpius* Jeram, 1994b (Mississippian [Visean] East Kirkton Quarry, West Lothian, Scotland) (Jeram 1990) and *Palaeoscorpius devonicus* Lehmann, 1944 (Early Devonian [Emsian] Hunsrück Slate, Rhineland-Palatinate, Germany) (Kühl et al. 2012). Book lungs are also present in an unnamed partial fossil from the Devonian (Emsian) Campbellton Formation (New Brunswick, Canada) (Shear et al. 1996), and *Eramoscorpius brucensis* Waddington et al., 2015 (Silurian [Wenlockian], Eramosa Formation, New York, USA) potentially also preserves book lungs (see discussion in Haug et al. 2019). Given the record within geologically older specimens, it is striking that book lungs are not known from later Palaeozoic specimens. This might reflect as-of-yet unconsidered taphonomic or recognition factors.

###  Palaeoecology

The elongated metasomal segment V (Fig. 2E) has implications for the biology of the Union Chapel Mine scorpion. Most Silurian, Devonian, and Carboniferous scorpions have metasomal segments of approximately the same length. This shifts later into the Carboniferous and through to today, with the evolution of an elongated 5th metasomal segment (Jeram 1994a). In modern forms, the 5th metasomal segment has different muscle groups to the preceding metasomal segments, and a hinge articulation with the telson that extends and lowers for stinging (Coelho et al. 2017; Günther et al. 2021). As longer metasomal segments are associated with longer, more rapidly contracting muscles (Coelho et al. 2017), the elongated segment would have permitted more rapid telson movement and a faster sting. This potentially reflects an adaptation for faster stinging ideal for deterring predators or catching rapidly moving prey. Indeed, the material here shows gracile chelae and a robust metasoma—morphologies commonly associated with utility of venom in prey capture and defence (Van der Meijden et al. 2013; Coelho et al. 2017) and illustrates an evolutionary trade-off between the two structures (Coelho et al. 2017). In modern scorpions, the gracile chelae and robust metasoma is common in Buthidae, a group that has evolved strong venom as a defence against mammalian predators (Santibáñez-López et al. 2022). Many defensive adaptations in Palaeozoic arthropods are thought to reflect responses to vertebrate predators (Shear and Kukalová-Peck 1990; Bicknell et al. 2022). We therefore propose that the elongated 5th metasomal segment represents a structure adapted to increased predation from animals such as amniotes, a group that increased in diversity during the Carboniferous and were abundant in the study area (e.g. Haubold et al. 2005; Long et al. 2025).

###  Life stage

The vast majority of fossil scorpions are moults (Jeram 1994b; Kjellesvig-Waering 1986; Stockwell 1989; McCoy and Brandt 2009). There are three significant criteria for differentiating a moult from a carcass (McCoy and Brandt 2009): the chelicerae are anteriorly extended, the body line is curved, and the legs are splayed for moults. These conditions are mostly reflected in the examined material here, with the exception of the legs that are poorly preserved. During moulting, the ventral plates can be rearranged or destroyed by the pectines (Kjellesvig-Waering 1986), and the exoskeleton separates anteriorly and laterally (Gaban and Farley 2002). The absence of coxosternal features likely reflects the moult condition. Such information is important for considering the development of this animal as modern scorpions moult between growth stages as juveniles (Gaban and Farley 2002). As such, this specimen represents an individual in a more juvenile stage.

###  Preservation

The fossil is completely carbonised (Figs. 3F and 4F) illustrating a preservational pathway lacking phosphatisation. The scorpion has therefore undergone kerogenisation and exoskeletal chitin was polymerised by thermal maturation into a long chain carbohydrate molecule (Gupta et al. 2006). Pyrolysis gas chromatography and mass spectrometry of two Carboniferous fossil scorpions from grey shales at the Lawrence Formation of the Lone Star Lake (Pennsylvanian [Kasimovian], Kansas, USA) and one specimen from the Joggins Formation (Pennsylvanian [Bashkirian], Nova Scotia, Canada) demonstrated that the fossils were preserved as aliphatic n-alk-1-enes and n-alkanes (hydrocarbons) (Stankiewicz et al. 1998; Abbott and Bashir 2021). Aliphatic hydrocarbons are composed entirely of hydrogen and carbon. The presence of this is indicated by the high abundance of carbon and low abundance of other elements in the Union Chapel Mine material. We therefore propose that the specimen passed through an in situ polymerisation of lipids, not the transport of hydrocarbons from surrounding sediments (Stankiewicz et al. 1998; Gupta et al. 2007). Living arthropod cuticle does not contain macromolecular aliphatic components. As such, the presence of these components within the fossil reflects diagenetic alteration (Briggs 1999). Experiments on fossilising scorpion cuticle demonstrated thermal maturation altered chitin into compounds including aliphatic hydrocarbons (Gupta et al. 2006).

Sulphur, calcium and phosphorus enrichment in the fossil is comparable to concentrations in the matrix. In other carbonaceous scorpion fossils from Carboniferous Coal Measures, sulphur has been interpreted as evidence for hyaline exocuticle (Bartram et al. 1987). We suggest that the lack of oxygen, and preservation of sulphur and calcium at similar abundances within the matrix evidence migration of elements from the surrounding sediment, not hyaline exocuticle. 

### Fluorescence 

Sternites and other cuticular structures fluoresced orange under UV light, while tergites did not react under UV light (Fig. 1A, C). Kerogen in coals fluoresces different colours depending on thermal maturity (Pradier et al. 1991; Senftle et al. 1993) with orange generally indicating intermediate thermal maturity (Senftle et al. 1993). As the fossil has undergone kerogenisation, this reaction under UV light is distinct from the exoskeletal fluorescence observed in modern forms. We demonstrate here that UV imagery is a useful tool for examining fossil scorpions, and this approach clearly illustrates boundaries of segments and other features like potential spiracles (Fig. 2G) that are less perceptible under standard visible light.

## Data Availability

No datasets were generated or analysed during the current study.
